# Supplementary material for: Contextualizing genetic risk score for disease screening and rare variant discovery
Source: Nat Commun. 2021 Jul 20;12:4418. doi: 10.1038/s41467-021-24387-z (PMC8292385; doi:10.1038/s41467-021-24387-z)
Supplement: Supplementary file 1 — Supplementary Information [file 41467_2021_24387_MOESM1_ESM.pdf]

Supplementary Information

Contextualizing genetic risk score for disease screening and rare variant discovery

Dan Zhou[1], Dongmei Yu[2,3], Jeremiah M. Scharf[2,3,4,5], Carol A. Mathews[6], Lauren McGrath[7], Edwin Cook[8], S. Hong Lee[9,10], Lea K. Davis[1, 11, 12, §], Eric R. Gamazon[1,13,14, §]

[1]Vanderbilt Genetics Institute; Division of Genetic Medicine, Department of Medicine, Vanderbilt University, Nashville, USA
[2]Psychiatric and Neurodevelopmental Genetics Unit, Center for Genomic Medicine, Department of Psychiatry, Massachusetts General Hospital, Boston, MA, USA
[3]Stanley Center for Psychiatric Research, Broad Institute of MIT and Harvard, Cambridge, MA, USA
[4]Department of Neurology, Massachusetts General Hospital, Boston, MA, USA
[5]Department of Neurology, Brigham and Women's Hospital, Boston, MA, USA
[6]Department of Psychiatry, Genetics Institute, University of Florida, Gainesville, FL, USA
[7]Department of Psychology, University of Denver, Denver, CO, USA
[8]Department of Psychiatry, Institute for Juvenile Research, University of Illinois at Chicago, Chicago, Illinois.
[9]Australian Centre for Precision Health, University of South Australia Cancer Research Institute, University of South Australia, Adelaide, SA, Australia
[10]UniSA Allied Health and Human Performance, University of South Australia, Adelaide, SA, Australia
[11]Department of Psychiatry and Behavioral Sciences, Vanderbilt University Medical Center, Nashville, TN, USA
[12]Department of Biomedical Informatics, Vanderbilt University Medical Center, Nashville, TN, USA
[13]Clare Hall, University of Cambridge, Cambridge, United Kingdom
[14]MRC Epidemiology Unit, University of Cambridge, Cambridge, United Kingdom

Send correspondence to:

Eric R. Gamazon <ericgamazon@gmail.com>
Lea K. Davis <lea.k.davis@vumc.org>

Supplementary Notes

The case-only study design

We compared the polygenic burden (PB) between large-effect variant (LEV) carriers and non-carriers among cases. We note that the test among cases is fundamentally different from an association test between PB and LEV. Higher PB among non-carriers than carriers could not be interpreted as a negative correlation between PB and LEV in the general population (a collider bias). In fact, PB and LEV are not correlated in the general population by study design. (We also examined the scenario in which the PB and LEV are dependent in a secondary analysis [see Results].) Generally speaking, collider bias can result in biased genetic associations or bias in associations between variables that influence study participation, and should be avoided[1, 2]. Spurious associations (e.g., genetic associations or associations between variables) can arise in the absence of true correlation in the intended study population[3]. However, in this study, our interest is elsewhere (i.e., *not* in inference on effect size in the intended study population).

Supplementary Table 1. The source of original studies involving the heritability estimates for all phenotypes. The estimated SNP-based heritability ranges from 0.25 (T1D) to 0.58 (TS).

Phenotype	Sample size case/control	Population prevalence	SNP-based heritability h^2 (se)	Original heritability reference
Tourette Syndrome (TS)	617/4,116	0.8%	0.58 (0.09)	Davis et al., 2013[12]
Obsessive Compulsive Disorder (OCD)	1,061/4,236	2.5%	0.37 (0.07)	Davis et al., 2013[12]
Type 1 Diabetes (T1D)	1,858/2,739	0.5%	0.25 (0.03)	WTCCC

Supplementary Table 2. Table describing samples and 'lrgCNV' events drawn from primary CNV publications. Qualifying CNVs were defined as large > 500 Kb, rare (< 1% DGV), and genic CNV events.

Phenotype	Number of Cases	Number of Controls	Number of Cases With lrgCNVs	Original CNV Publication
Tourette Syndrome (TS)§	1,086	1,789	77 (7%)	McGrath et al., 2014[13]
Obsessive Compulsive Disorder (OCD)§	1,613	1,789	88 (5%)	McGrath et al., 2014[13]

§Denotes the shared controls between TS and OCD samples.

Supplementary Table 3. Relationship between common-variant polygenic load and rare variant status within case samples representing three complex traits.

Phenotypic Classification	N cases (%large CNV)	Variance explained	Wilcoxon Rank Sum Test (p-value)
Tourette Syndrome (TS)	516 (6.9%)	3.6%	0.020
Obsessive Compulsive Disorder (OCD)	919 (6.6%)	0.4%	0.120
Type 1 Diabetes (T1D)	1,858 (47%)	0.5%	0.007

'Phenotypic classification' defines the case sample used for each analysis presented in the table. For TS and OCD, we created the phenotype lrgCNV carrier and annotated those individuals carrying at least one qualifying CNV event (large, rare, genic CNVs). 'N cases' is the total number of cases (with lrgCNV measured) included in the regression models. The proportion of lrgCNV carriers is provided in parentheses. For T1D, we annotated cases based on the number of known high-risk genotypes at the HLA-*DRB1* locus[14] (high-risk genotypes = 3/3, 3/4, or 4/4 alleles; lower risk genotypes = 3/not [3 or 4], 4/not [3 or 4], or not [3 or 4]/not [3 or 4]). For T1D, the proportion of high-risk HLA-*DRB1* is provided in parentheses. 'Variance explained' is the proportion of variance in lrgCNV carrier status (for TS and OCD) and in number of HLA-*DRB1* risk alleles (for T1D) explained by the PGS. For TS and OCD we calculated the difference in Nagelkerke's pseudo R^2 between the full model and a reduced model that excluded the PGS term. For T1D we report the R^2 from linear regression on the number of high-risk alleles present. 'Wilcoxon rank sum test (p-value)' provides the p-value for the (one-sided) non-parametric test of lower adjusted PGS group means in lrgCNV carriers than in non-carriers for TS and OCD. For T1D, we provide the p-value for the non-parametric test of adjusted PGS group means between HLA-*DRB1* high-risk homozygotes compared to heterozygotes and low-risk homozygotes.

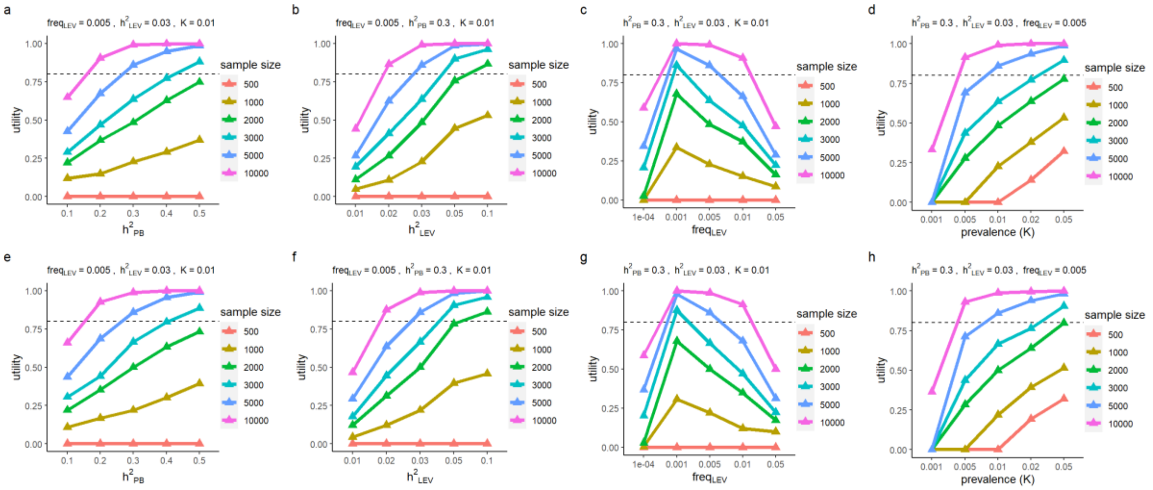

Supplementary Figure 1. Utility under liability-threshold model. Similar to Figure 3, we calculated the utility under two additional genetic architectures, i.e. (a-d) negative selection and (e-h) LD-adjusted kinship models. For each panel, we varied one parameter (x-axis) while fixing the other parameters (top of each panel). Broken line at 80% is a reasonable utility threshold. Source data are provided as a Source Data file.

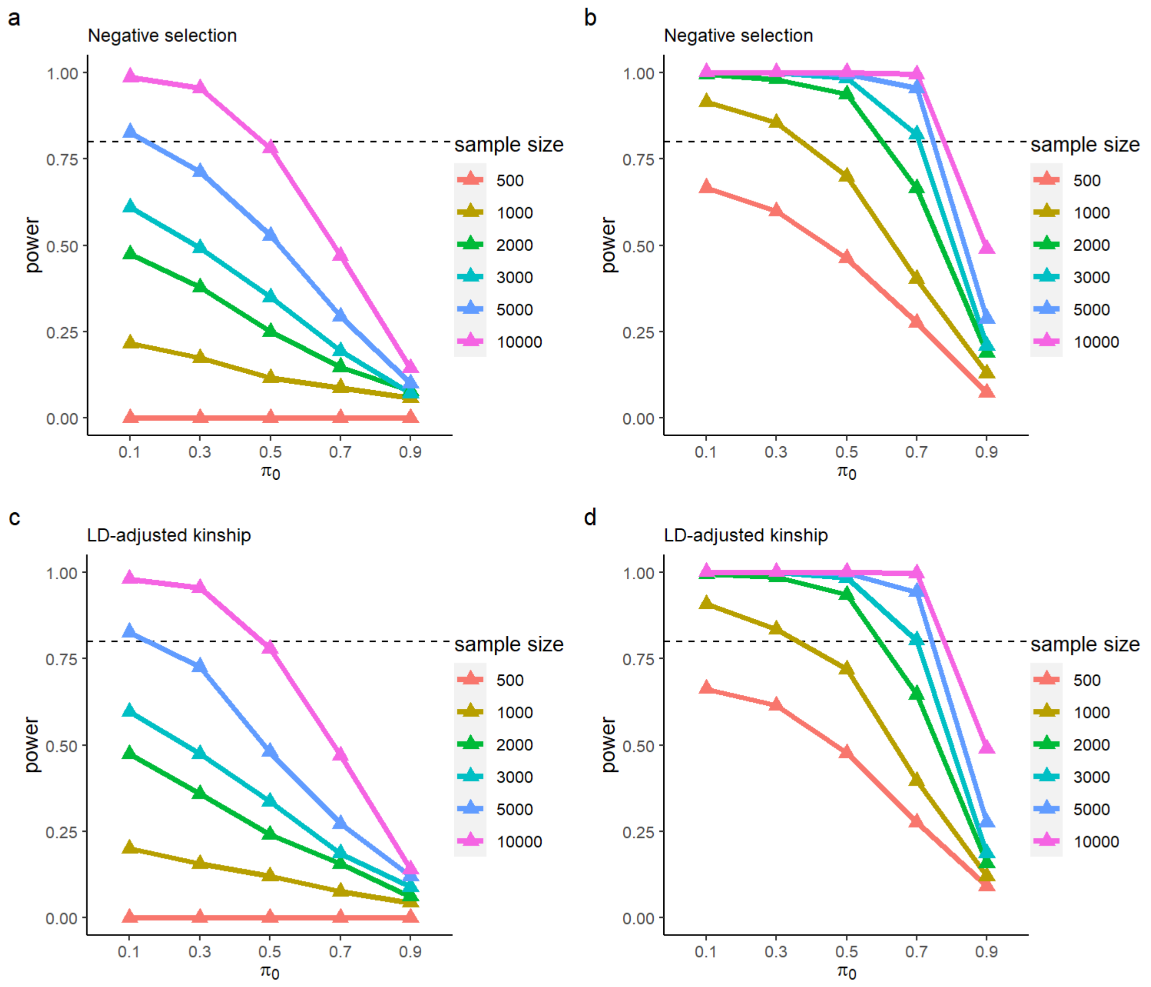

Supplementary Figure 2. Power estimation under liability-threshold model. Similar to Figure 4, we estimated the power under two additional genetic architectures, i.e. (a, b) negative selection and (c, d) LD-adjusted kinship models and two scenarios (1) left two panels(a and c, $h^2_{PB} = 0.3, h^2_{LEV} = 0.03, freq_{LEV} = 0.005, K = 0.01$), and (2) right two panels (b and d, $h^2_{PB} = 0.5, h^2_{LEV} = 0.1, freq_{LEV} = 0.05, K = 0.05$). Broken line at 80% is a reasonable threshold for power. Source data are provided as a Source Data file.

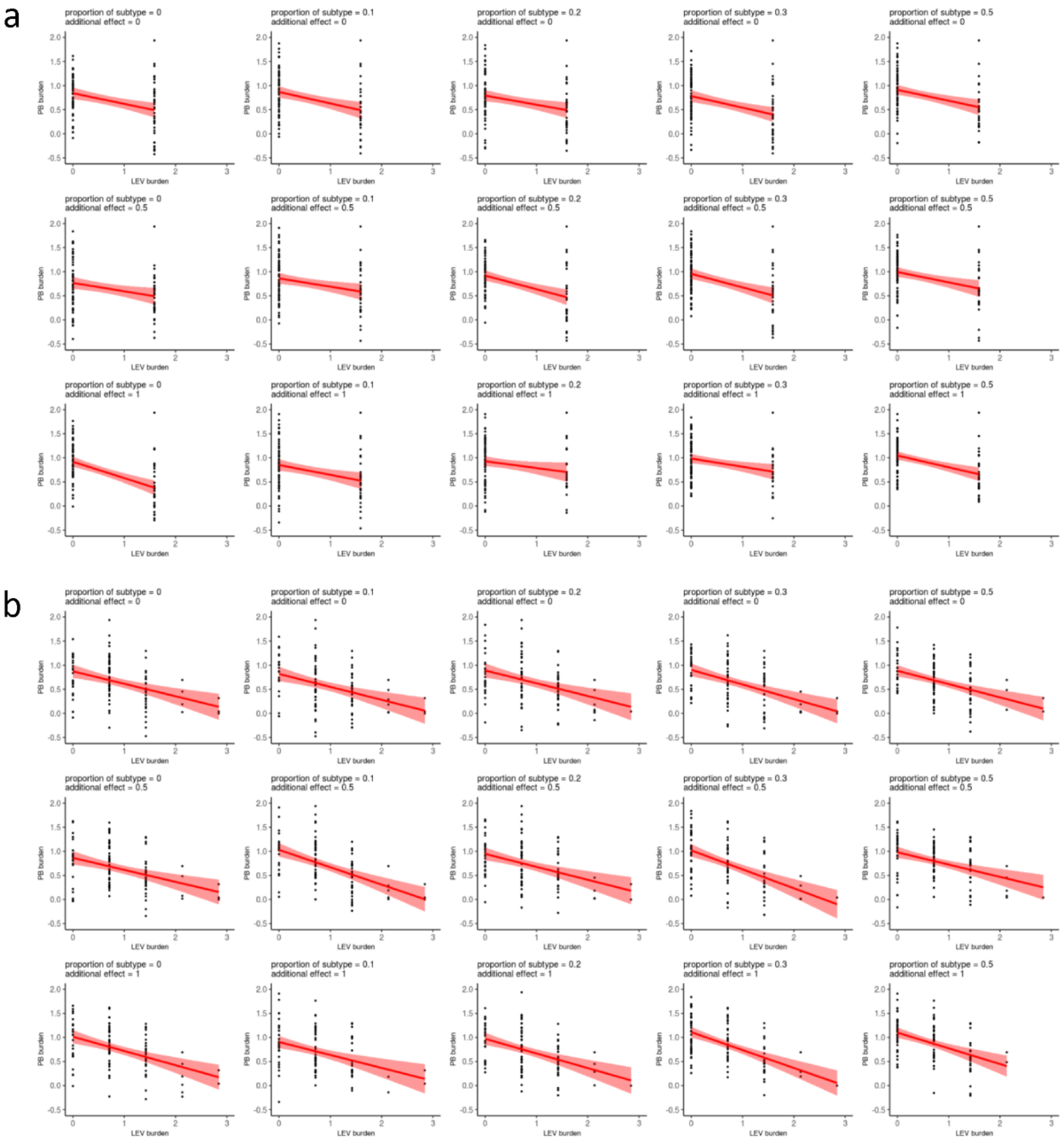

Supplementary Figure 3. The PB-LEV relationship in the presence of disease subtypes. Using the liability-threshold model and polygenic genetic architecture, we assumed 1% of the samples (N_{total} = 10,000) are cases. For the cases, the LEV burden (R) and the PB (A) are displayed on x-axis and y-axis, respectively. Panel (a) and panel (b) assume one LEV and ten independent LEVs (MAF = 0.01), respectively. Here, we assumed two subtypes, namely, *major* and *minor*. The heterogeneous effect (λ; see Methods) in the subjects with the *minor* subtype and the proportion of cases with the *minor* subtype are reflected in the rows and columns, respectively. A linear regression line (red) was fitted for each sub-panel, with the 95% confidence interval shown. Source data are provided as a Source Data file.

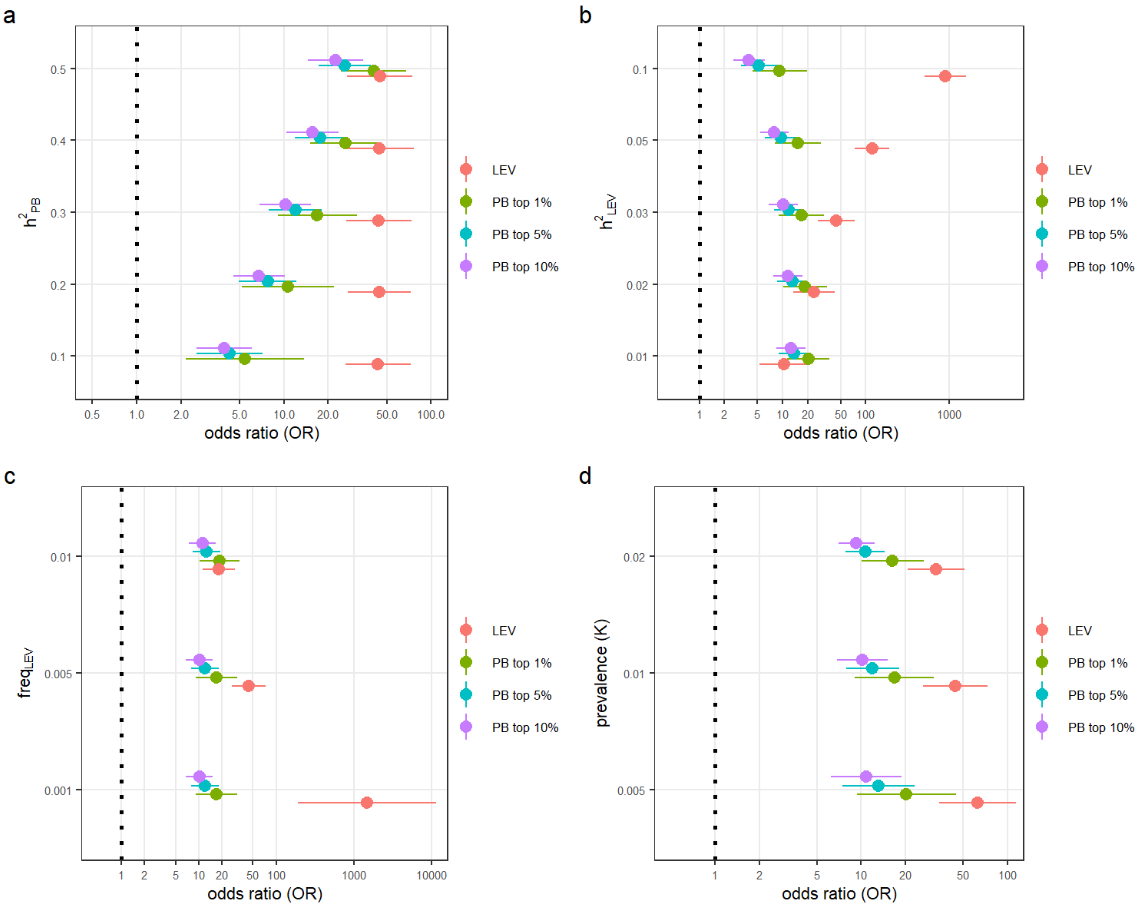

Supplementary Figure 4. Odds ratio (OR) comparison between the LEV and the PB under a genetic architecture consistent with negative selection and liability-threshold model of disease risk. Similar to Figure 6, we calculated the OR for LEV and PB while varying the parameters. The point estimates and the 95% confidence interval (CI) are shown as dots and horizontal lines, respectively. The vertical broken line at OR = 1 shows the null. In panel (a), we fixed h^2_{LEV}, f, and K at 0.03, 0.005, and 0.01, respectively, while varying h^2_{PB}. In panel (b), we fixed h^2_{PB}, f, and K at 0.3, 0.005, and 0.01, respectively, while varying h^2_{LEV}. In panel (c), we fixed h^2_{PB}, h^2_{LEV}, and K at 0.3, 0.03, and 0.01, respectively, while varying f. In panel (d), we fixed h^2_{PB}, h^2_{LEV}, and f at 0.3, 0.03, and 0.005, respectively, while varying K. Source data are provided as a Source Data file.

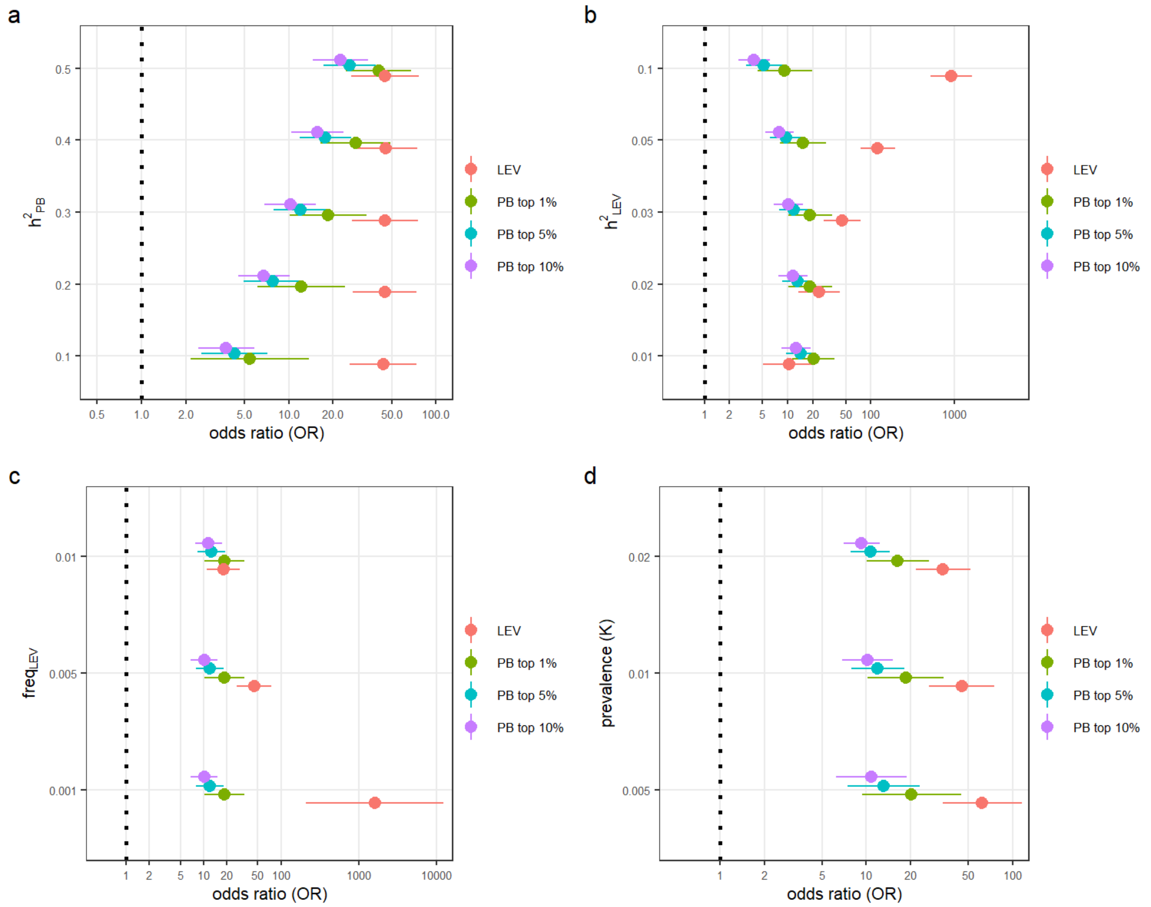

Supplementary Figure 5. Odds ratio (OR) comparison between the LEV and the PB under LD-adjusted kinship based genetic architecture and liability-threshold model of disease risk. Similar to Figure 6, we calculated the OR for LEV and PB while varying the parameters. The point estimates and the 95% confidence interval (CI) are shown as dots and horizontal lines, respectively. The vertical broken line at OR = 1 shows the null. In panel (a), we fixed h^2_{LEV}, f, and K at 0.03, 0.005, and 0.01, respectively, while varying h^2_{PB}. In panel (b), we fixed h^2_{PB}, f, and K at 0.3, 0.005, and 0.01, respectively, while varying h^2_{LEV}. In panel (c), we fixed h^2_{PB}, h^2_{LEV}, and K at 0.3, 0.03, and 0.01, respectively, while varying f. In panel (d), we fixed h^2_{PB}, h^2_{LEV}, and f at 0.3, 0.03, and 0.005, respectively, while varying K. Source data are provided as a Source Data file.

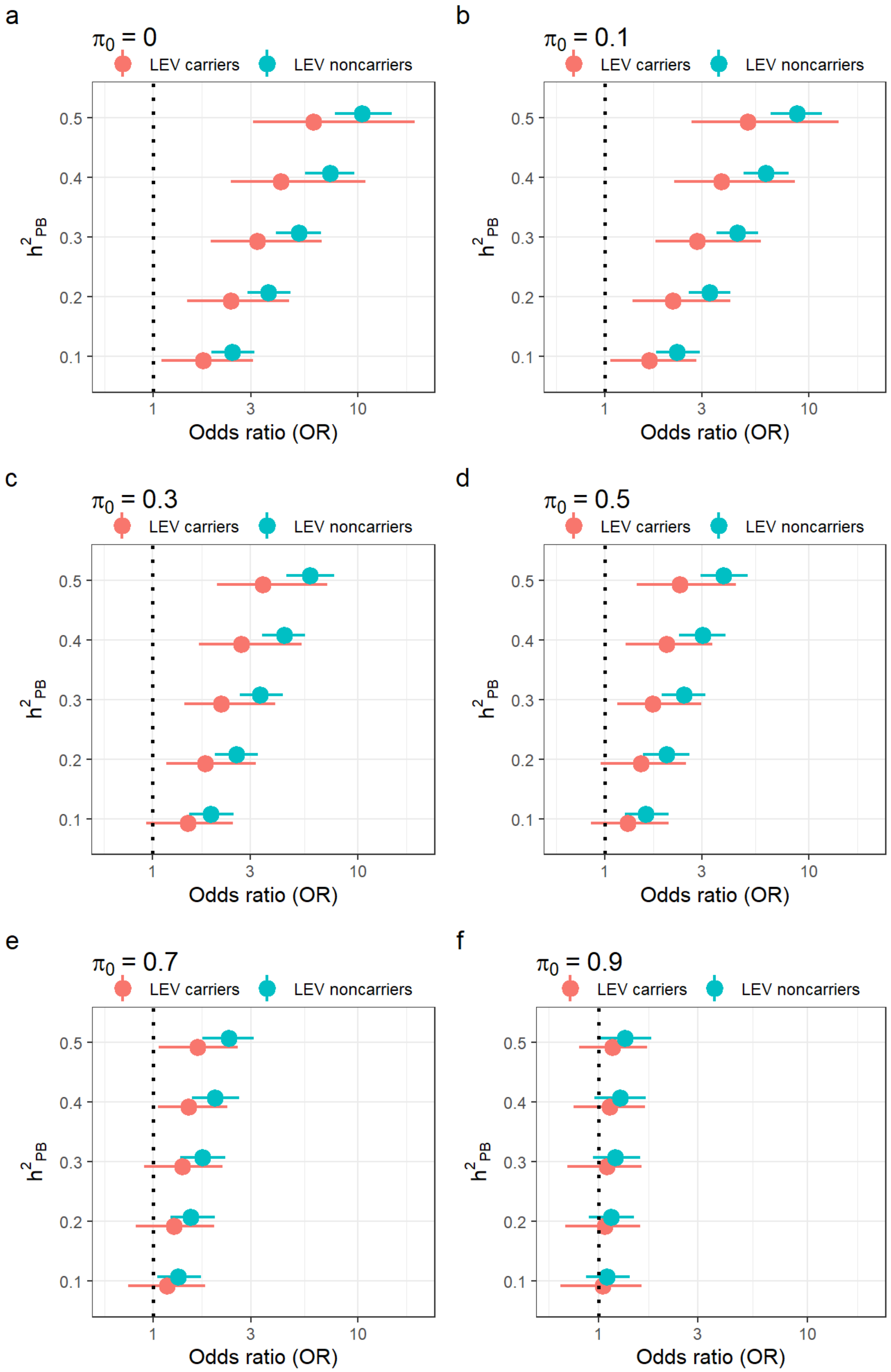
a
$\pi_0 = 0$
b
$\pi_0 = 0.1$
c
$\pi_0 = 0.3$
d
$\pi_0 = 0.5$
e
$\pi_0 = 0.7$
f
$\pi_0 = 0.9$
LEV carriers
LEV noncarriers
h^2_{PB}
0.1
0.2
0.3
0.4
0.5
1
3
10
Odds ratio (OR)

Supplementary Figure 6. Comparison of the odds ratio (OR) of the PB between LEV carriers and non-carriers while varying the proportion of non-causal variants (π_0). In these simulations, we fixed h^2_{LEV}, f, and K at 0.03, 0.005, and 0.01, respectively, while varying π_0 and h^2_{PB}. The OR of the PB was estimated under the liability-threshold model while varying the proportion of non-causal variants (π_0) from 0 to 0.9 (panel a to panel e). In each panel, we also varied the common SNP-based heritability (h^2_{PB}) from 0.1 to 0.5. The OR of the PB was estimated in LEV carriers and in non-carriers separately. In simulations, we assumed 10,000 samples. The median of the OR across simulations is shown as a dot, while the P2.5 and P97.5 quantiles of the OR across simulations are represented by the horizontal segments. Source data are provided as a Source Data file.

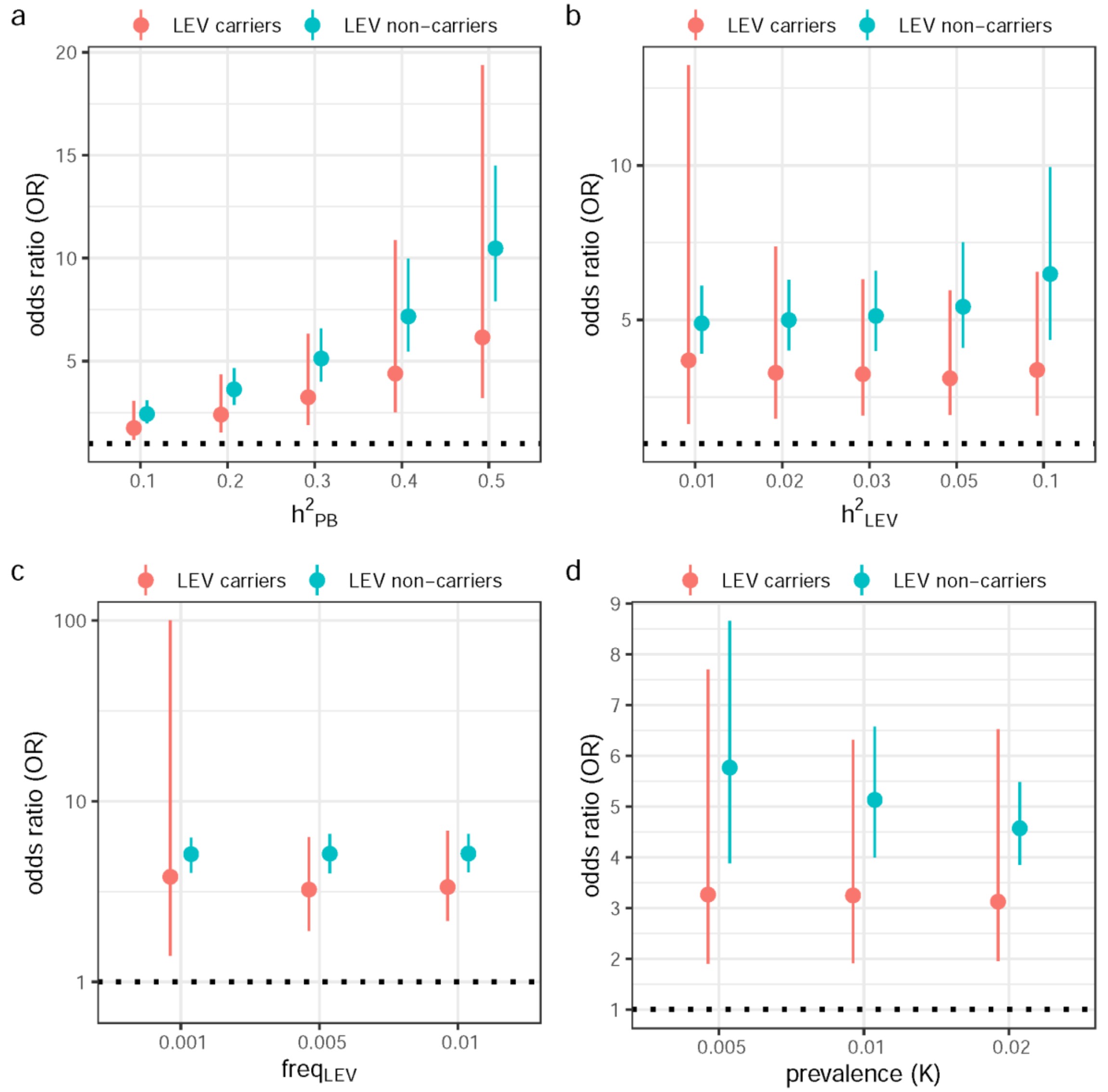

Supplementary Figure 7. Change in the OR of PB (per sd change) with respect to change in parameter differs between LEV carriers and non-carriers. The OR of the PB was calculated under a genetic architecture consistent with negative selection while varying the common SNP-based heritability (h^2_{PB}), the heritability of LEV (h^2_{LEV}), the allele frequency of LEV(f), and the prevalence (K). In simulations, we assumed 10,000 samples. The median of the OR across simulations is shown as a dot, while the 2.5th and 97.5th percentile of the OR across simulations are represented by the horizontal segments. The results under the polygenic model are presented here, as the same pattern was found for the other genetic architecture models. In panel (a), we fixed h^2_{LEV}, f, and K at 0.03, 0.005, and 0.01, respectively, while varying h^2_{PB}. In panel (b), we fixed h^2_{PB}, f, and K at 0.3, 0.005, and 0.01, respectively, while varying h^2_{LEV}. In panel (c), we fixed h^2_{PB}, h^2_{LEV}, and K at 0.3, 0.03, and 0.01, respectively, while varying f. In panel (d), we fixed h^2_{PB}, h^2_{LEV}, and f at 0.3, 0.03, and 0.005, respectively, while varying K. Among the parameters tested here, the h^2_{PB} is the most important determinant of how differently, between carriers and non-carriers, the OR of the PB changes, as can be seen from the "slope" at each point. Source data are provided as a Source Data file.

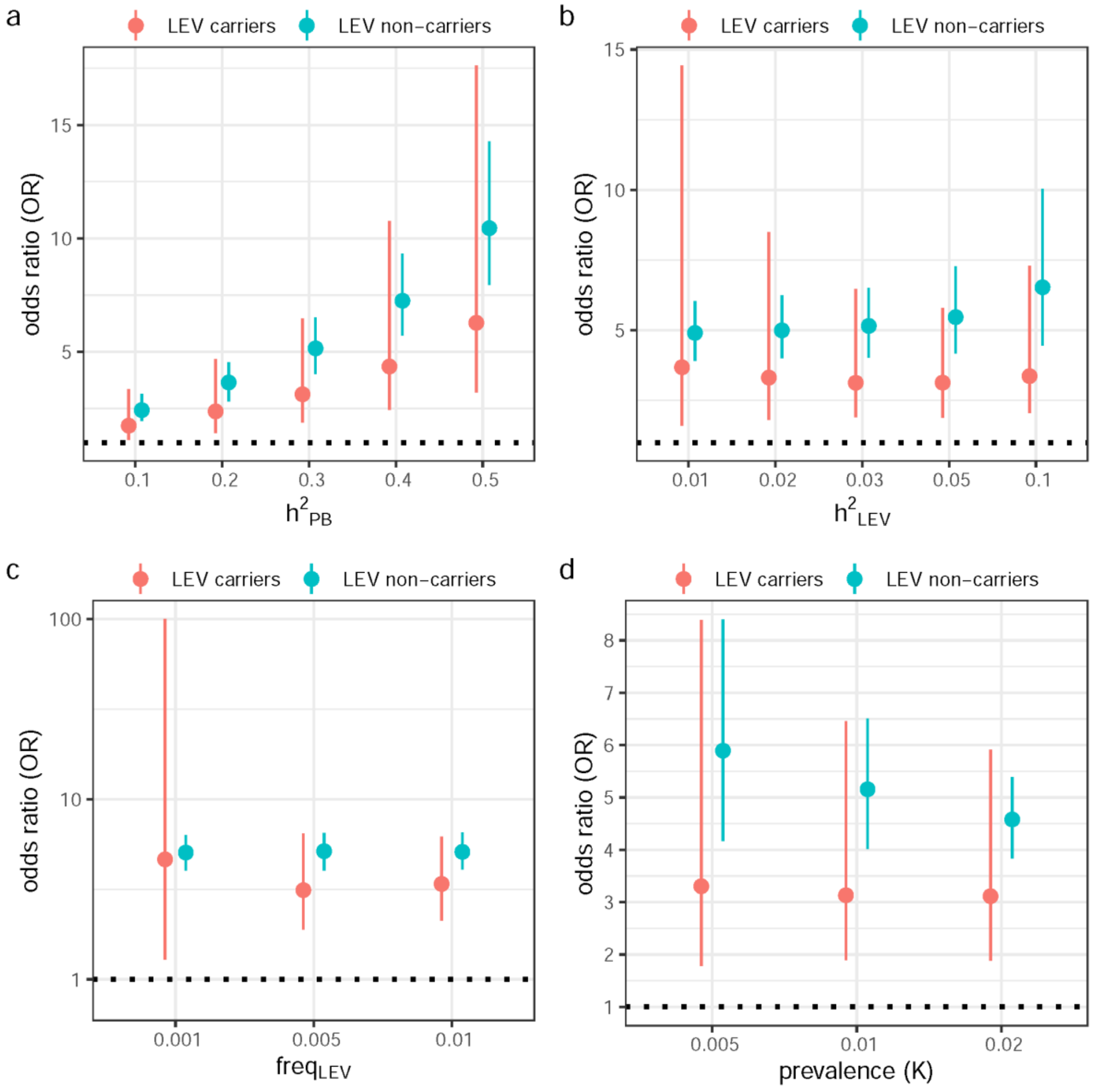

Supplementary Figure 8. Change in the OR of PB (per sd change) with respect to change in parameter differs between LEV carriers and non-carriers. The OR of the PB was calculated under the LD-adjusted kinship based genetic architecture while varying the common SNP-based heritability (h^2_{PB}), the heritability of LEV (h^2_{LEV}), the allele frequency of LEV(f), and the prevalence (K). In simulations, we assumed 10,000 samples. The median of the OR across simulations is shown as a dot, while the 2.5th and 97.5th percentile of the OR across simulations are represented by the horizontal segments. The results under the polygenic model are presented here, as the same pattern was found for the other genetic architecture models. In panel (a), we fixed h^2_{LEV}, f, and K at 0.03, 0.005, and 0.01, respectively, while varying h^2_{PB}. In panel (b), we fixed h^2_{PB}, f, and K at 0.3, 0.005, and 0.01, respectively, while varying h^2_{LEV}. In panel (c), we fixed h^2_{PB}, h^2_{LEV}, and K at 0.3, 0.03, and 0.01, respectively, while varying f. In panel (d), we fixed h^2_{PB}, h^2_{LEV}, and f at

0.3, 0.03, and 0.005, respectively, while varying K. Among the parameters tested here, the h_{PB}^2 is the most important determinant of how differently, between carriers and non-carriers, the OR of the PB changes, as can be seen from the "slope" at each point. Source data are provided as a Source Data file.

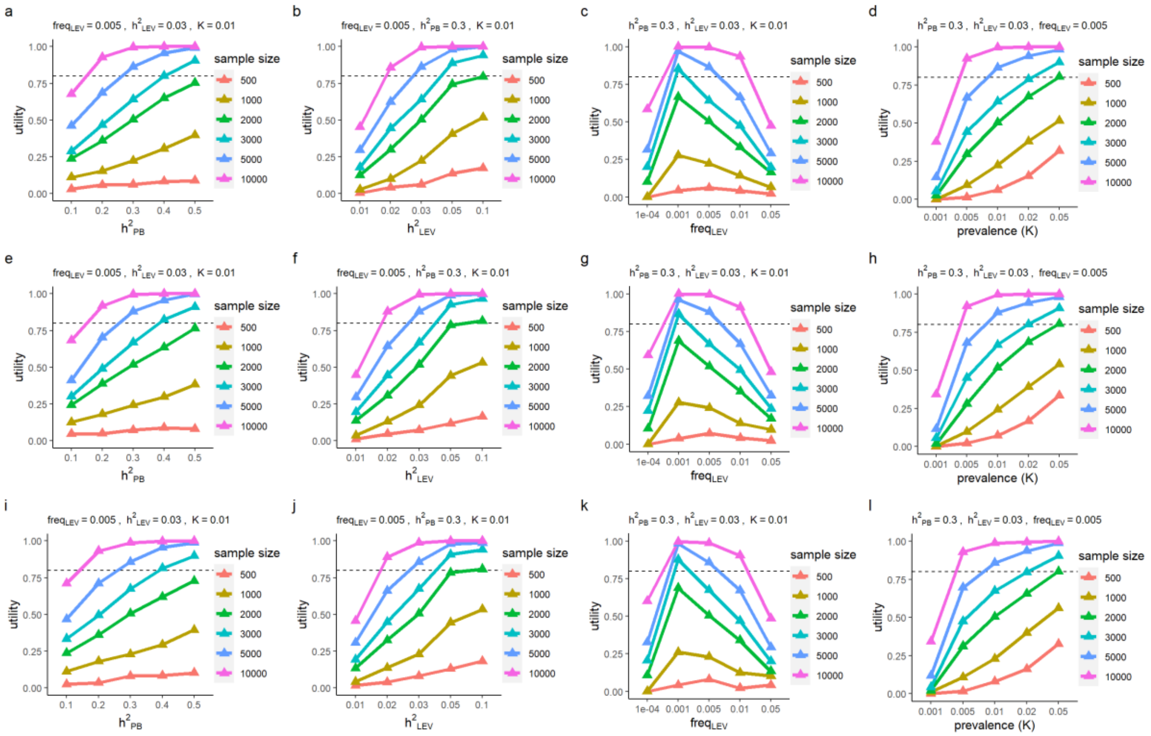

Supplementary Figure 9. Utility of PB-LEV correlation under logit risk model. We simulated the effect sizes for common and rare variants and calculated the probability of disease risk for each individual by a logit link function. Cases were stochastically drawn from the binomial distribution using the probability of disease risk. For each column, we varied the heritability attributable to the common-variant polygenic component, the heritability captured by the LEV, and its allele frequency while fixing all other parameters. For each row, we simulated the effect sizes for common variants under different genetic architectures. Two-sample Wilcoxon test was performed to test whether the PB burden was lower in LEV carriers than in non-carriers (one-sided test) in cases. The utility was calculated as the proportion of significant (P < 0.05) test results in 500 simulations with different seeds for sampling causal variants and subjects. Broken line at 80% is a reasonable utility threshold. Source data are provided as a Source Data file.

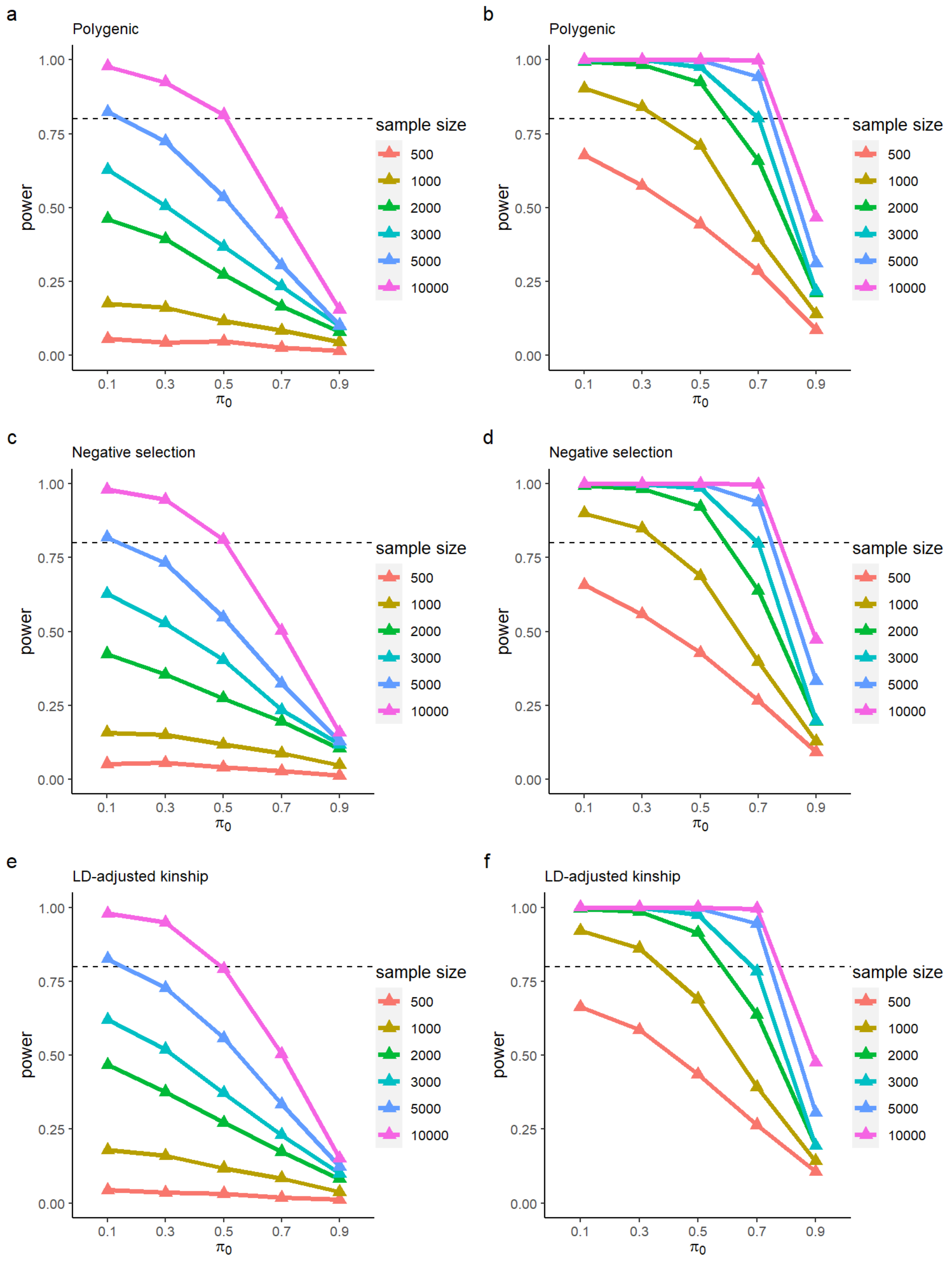

Supplementary Figure 10. Power under logit risk model. The proportion π_0 (0.1-0.9) of non-causal common variants in the polygenic component was varied in power estimation. For the other parameters, three different combinations were applied. Under three genetic architect assumptions (polygenic [panel a and b],

negative selection [panel c and d], and LD-adjusted kinship [panel e and f]), we simulated two combinations of parameters including (1) ‘lower level’ (panel a, c, and e) with $h^2_{PB} = 0.3, h^2_{LEV} = 0.03, f = 0.005$, prevalence $(K) = 0.01$, and (2) ‘higher level’ (panel b, d, and f) with $h^2_{PB} = 0.5, h^2_{LEV} = 0.1, f = 0.05$, prevalence $(K) = 0.05$ to show the effect of π_0 on the power at different levels. Broken line at 80% is a reasonable threshold for power. Source data are provided as a Source Data file.

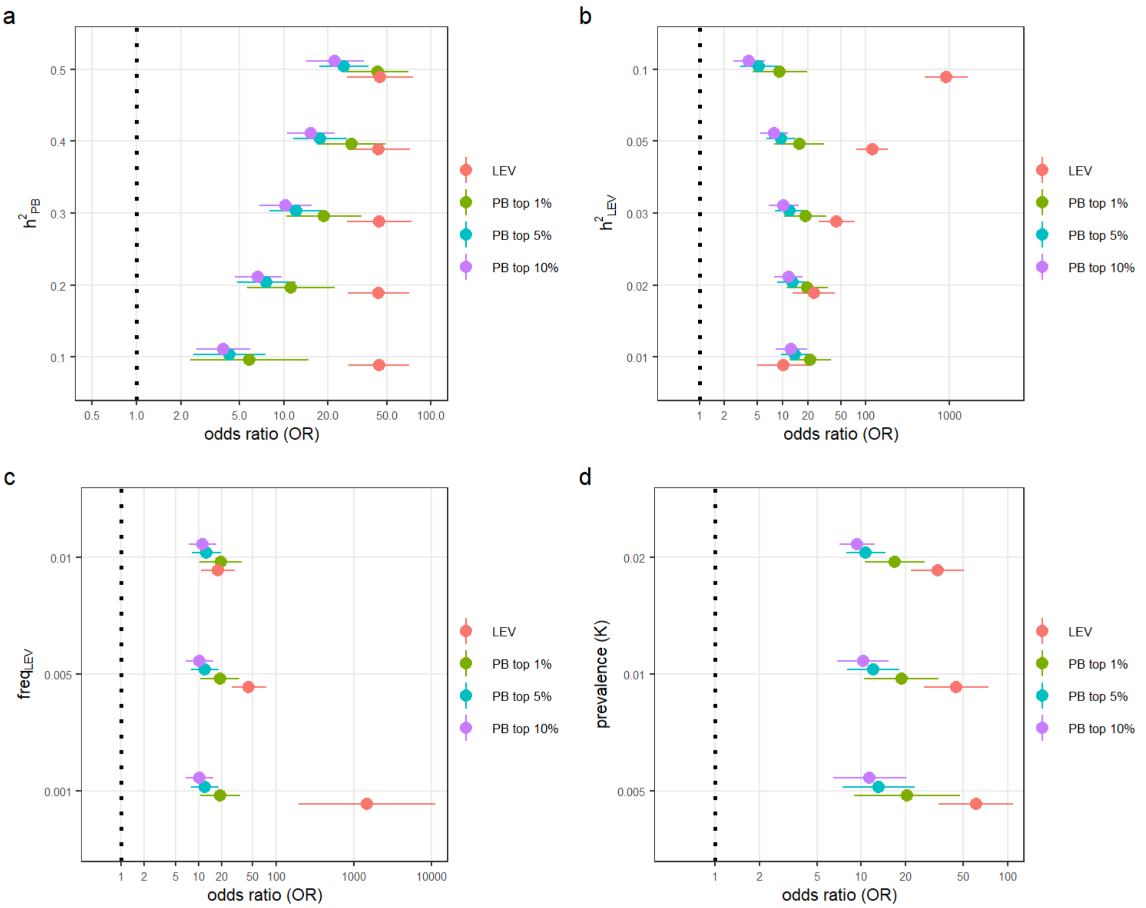

Supplementary Figure 11. Odds ratio (OR) comparison between the LEV and the PB under the polygenic genetic architecture model and logit risk model of disease risk. The OR of the LEV was calculated under the logit risk model while varying the common SNP-based heritability, heritability of LEV, allele frequency of LEV, and the prevalence. The OR of the polygenic burden was calculated by comparing individuals with high PB (top 1%, 5%, 10% of distribution) with the remainder of the population. The point estimates and the 95% confidence interval

(CI) are shown as dots and horizontal lines, respectively. Source data are provided as a Source Data file.

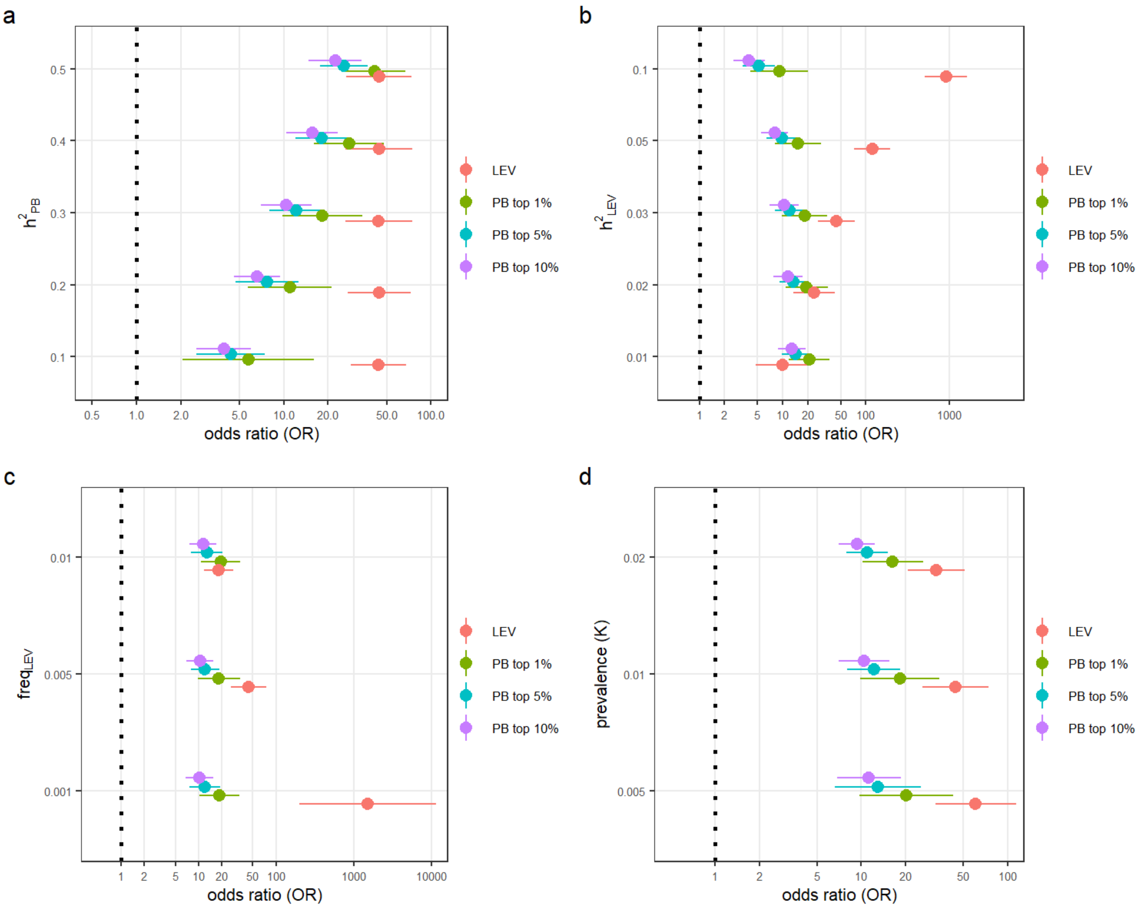

Supplementary Figure 12. Odds ratio (OR) comparison between the LEV and the PB under a genetic architecture consistent with negative selection and logit risk model of disease risk. The OR of the LEV was calculated under the logit risk model while varying the common SNP-based heritability, heritability of LEV, allele frequency of LEV, and the prevalence. The OR of the polygenic burden was calculated by comparing individuals with high PB (top 1%, 5%, 10% of distribution) with the remainder of the population. The point estimates and the 95% confidence interval (CI) are shown as dots and horizontal lines, respectively. Source data are provided as a Source Data file.

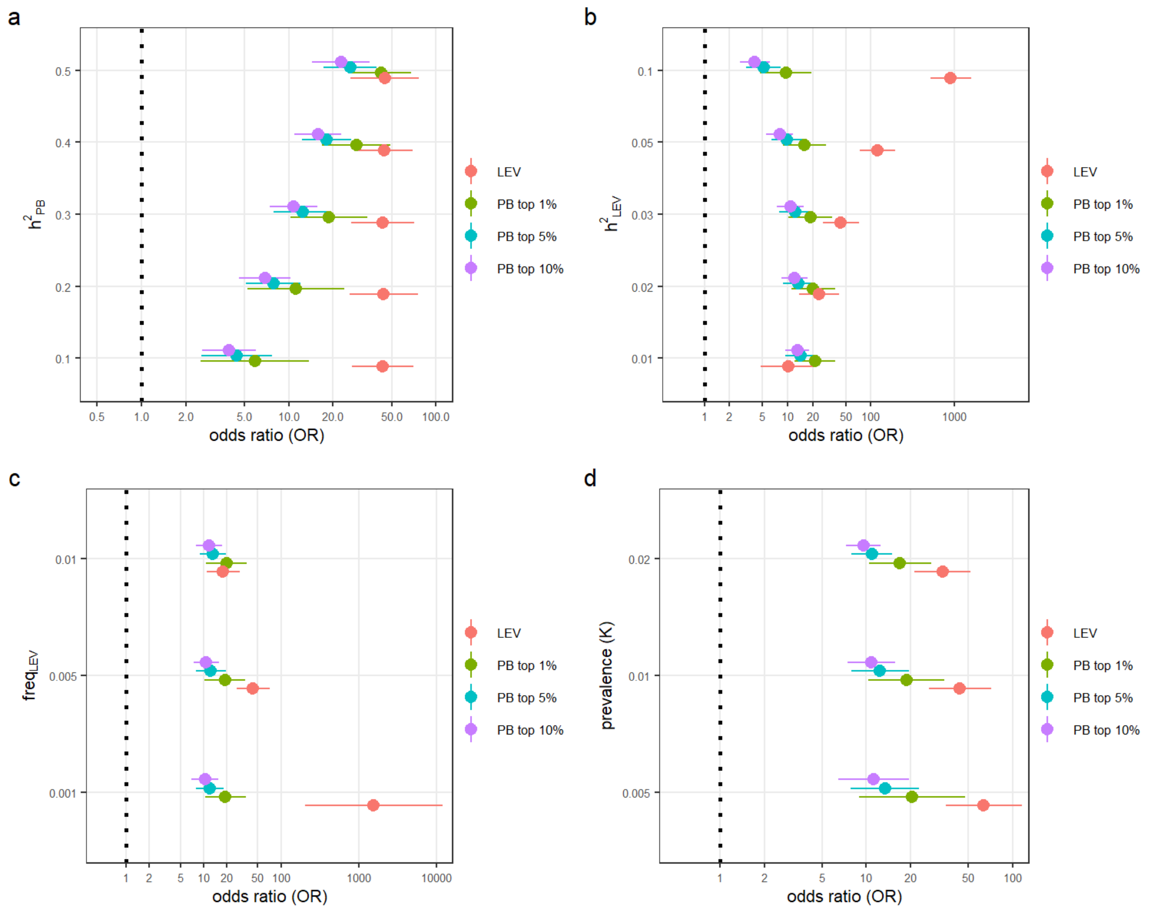

Supplementary Figure 13. Odds ratio (OR) comparison between the LEV and the PB under LD-adjusted kinship genetic architecture and logit risk model of disease risk. The OR of the LEV was calculated under the logit risk model while varying the common SNP-based heritability, heritability of LEV, allele frequency of LEV, and the prevalence. The OR of the polygenic burden was calculated by comparing individuals with high PB (top 1%, 5%, 10% of distribution) with the remainder of the population. The point estimates and the 95% confidence interval (CI) are shown as dots and horizontal lines, respectively. Source data are provided as a Source Data file.

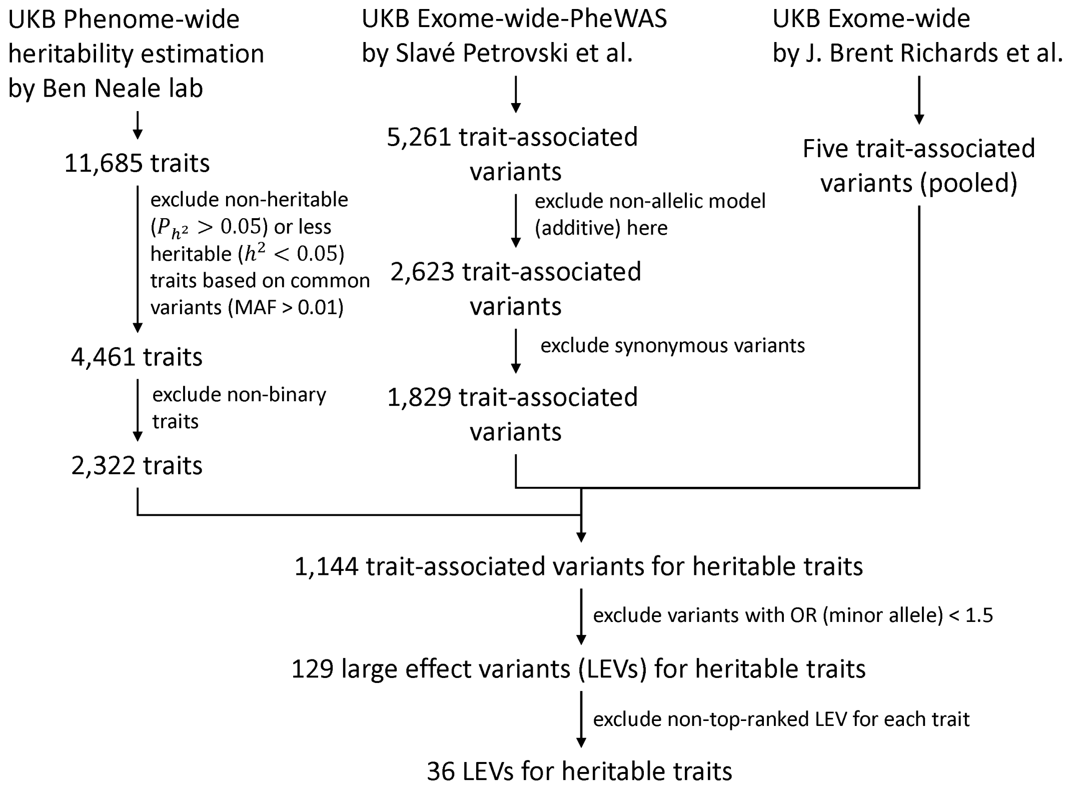

Supplementary Figure 14. The flowchart for processing summary-statistic-level data from large-scale biobank (the UK Biobank). The common-variant-based heritability was estimated from chip-based SNPs (Neale Lab). The effect size and MAF of the LEV were estimated from a variant-level exome-wide association study in the UK Biobank samples. The simulation framework was developed based on the empirical parameters.

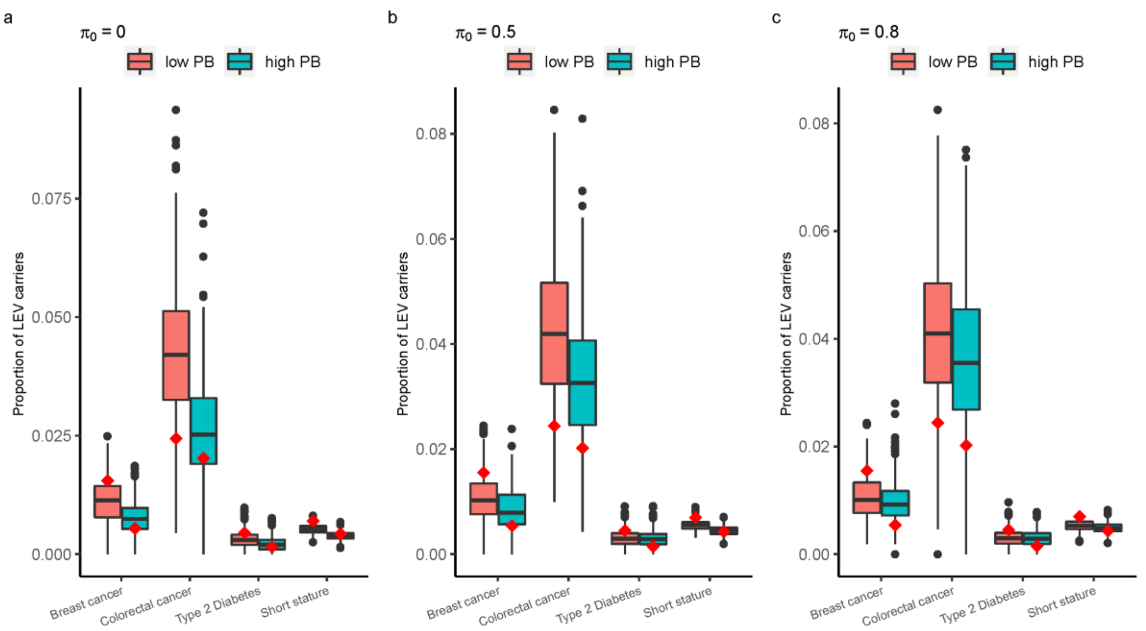

Supplementary Figure 15. Prediction of the framework on proportion of LEV carriers in cases with a given PB profile matches empirical observations, providing an LEV screening approach (logit risk model). Using empirically derived parameters (including the common variant based heritability, disease prevalence, allele frequency, and odds ratio of the LEV) estimated from UK Biobank, we performed simulations for four traits under the genetic architecture in line with negative selection. We varied the π_0 (the proportion of non-causal variants in the polygenic risk score) at (a) 0, (b) 0.5, and (c) 0.8. Cases were defined under the liability-threshold model and classified into 'low-PB' and 'high-PB' (using the median as the cutoff) groups. The proportion of LEV carriers was defined as the number of cases carrying LEV over the total number of cases in each group. The distribution of the proportion (among 500 simulated sets) is shown in the boxplot. The median of the proportion is visualized as a black segment in the middle of the box. The lower and upper hinges correspond to the first and third quartiles (the 25th and 75th percentiles). The upper / lower whisker extends from the hinge to the largest / smallest value no further than/ at most 1.5 * IQR from the hinge (where IQR is the inter-quartile range or the distance between the first and third quartiles). The actual observed proportion of LEV carriers for each PB profile from empirical data (with the matching parameters as the simulations) is marked as a red diamond. Thus, the prediction of the framework and the empirical dataset (with matching parameters as the simulations) were concordant. Source data are provided as a Source Data file.

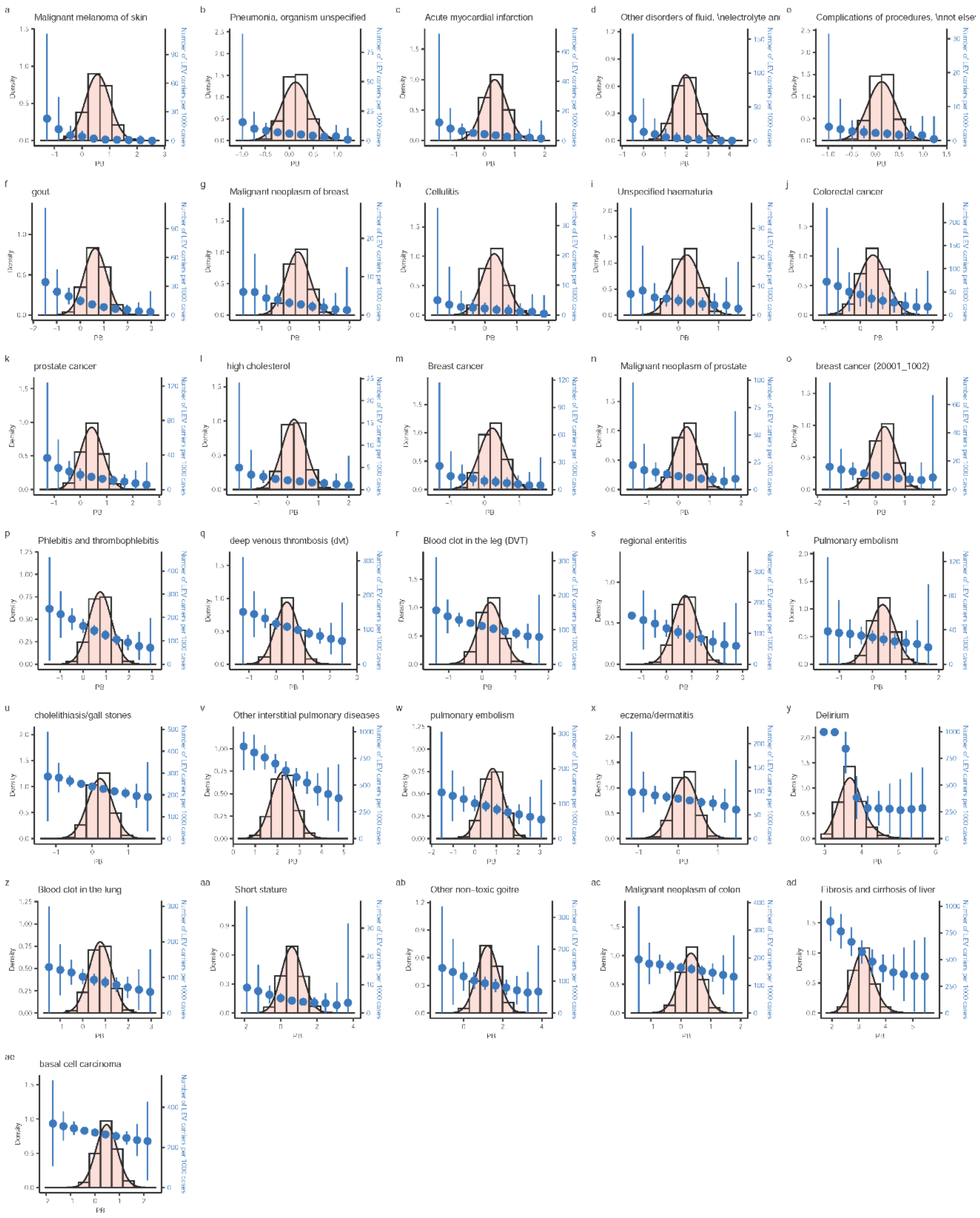

Supplementary Figure 16. Cases with low polygenic risk score have higher probability of carrying an LEV. Based on empirically derived parameters from the UK Biobank, we performed simulations (under the liability-threshold model and the genetic architecture in line with negative selection) and compared the number of LEV carriers per 1000 cases among the different polygenic risk scores for 31 UK Biobank traits (additional to Figure 7). For each trait, we grouped the cases into 10

equally-sized polygenic risk score bins. For each bin, the mean ± 1sd of the number of LEV-carriers per 1000 cases is displayed in blue circles and bars. The distribution of polygenic risk score is shown as a histogram. Thus, the framework (assuming pre-specified simulation parameters) provides testable predictions on the number of LEV carriers per 1000 cases with a given PB profile and on the sample polygenic risk score profile to optimize LEV screening. Detailed results can be found in Supplementary Data 2.

REFERENCES

1. Day FR, Loh P-R, Scott RA, Ong KK, Perry JR. A robust example of collider bias in a genetic association study. *The American Journal of Human Genetics* **98**, 392-393 (2016).

2. Aschard H, Vilhjálmsson BJ, Joshi AD, Price AL, Kraft P. Adjusting for heritable covariates can bias effect estimates in genome-wide association studies. *The American Journal of Human Genetics* **96**, 329-339 (2015).

3. Munafò MR, Tilling K, Taylor AE, Evans DM, Davey Smith G. Collider scope: when selection bias can substantially influence observed associations. *Int J Epidemiol* **47**, 226-235 (2017).

4. Cross-Disorder Group of the Psychiatric Genomics C*, et al.* Genetic relationship between five psychiatric disorders estimated from genome-wide SNPs. *Nature genetics* **45**, 984-994 (2013).

5. Davis LK*, et al.* Partitioning the heritability of Tourette syndrome and obsessive compulsive disorder reveals differences in genetic architecture. *PLoS genetics* **9**, e1003864 (2013).

6. Yang J, Lee SH, Goddard ME, Visscher PM. GCTA: a tool for genome-wide complex trait analysis. *American journal of human genetics* **88**, 76-82 (2011).

7. McGrath LM*, et al.* Copy number variation in obsessive-compulsive disorder and tourette syndrome: a cross-disorder study. *Journal of the American Academy of Child and Adolescent Psychiatry* **53**, 910-919 (2014).

8. Sanders SJ*, et al.* Multiple recurrent de novo CNVs, including duplications of the 7q11.23 Williams syndrome region, are strongly associated with autism. *Neuron* **70**, 863-885 (2011).

9. Szatkiewicz JP*, et al.* Detecting large copy number variants using exome genotyping arrays in a large Swedish schizophrenia sample. *Molecular psychiatry* **18**, 1178-1184 (2013).

10. Noble JA, Valdes AM, Cook M, Klitz W, Thomson G, Erlich HA. The role of HLA class II genes in insulin-dependent diabetes mellitus: molecular analysis of 180 Caucasian, multiplex families. *American journal of human genetics* **59**, 1134 (1996).

11. Howson JM, Walker NM, Clayton D, Todd JA, Type 1 Diabetes Genetics C. Confirmation of HLA class II independent type 1 diabetes associations in the major histocompatibility complex including HLA-B and HLA-A. *Diabetes Obes Metab* **11 Suppl 1**, 31-45 (2009).

12. Davis LK*, et al.* Partitioning the heritability of Tourette syndrome and obsessive compulsive disorder reveals differences in genetic architecture. *PLoS genetics* **9**, (2013).

13. McGrath LM*, et al.* Copy number variation in obsessive-compulsive disorder and tourette syndrome: a cross-disorder study. *Journal of the American Academy of Child & Adolescent Psychiatry* **53**, 910-919 (2014).

14. Noble JA, Valdes AM. Genetics of the HLA region in the prediction of type 1 diabetes. *Current diabetes reports* **11**, 533 (2011).
