# Supplementary material for: Contextualizing genetic risk score for disease screening and rare variant discovery
Source: Nat Commun. 2021 Jul 20;12:4418. doi: 10.1038/s41467-021-24387-z (PMC8292385; doi:10.1038/s41467-021-24387-z)
Supplement: Supplementary file 3 — Description of Additional Supplementary Files [file 41467_2021_24387_MOESM3_ESM.pdf]

Description of Additional Supplementary Files

Supplementary Data 1: Polygenic burden comparison (t-test) between simulated cases and controls

A two-sample t-test was used to test if the polygenic burden is higher in cases than in controls (one-sided test). We assumed polygenic genetic architecture and varied other parameters including the heritability due to polygenic burden (h2_PB), the heritability due to large-effect variant (h2_LEV), the minor allele frequency of LEV (freq_LEV), the prevalence, sample size, and the disease model.

Supplementary Data 2: Summary-statistics and prediction results from the UK Biobank

In columns A-R, we summarized the empirical data from multiple UK Biobank-based sources. Based on the parameters estimated from the UK Biobank, we estimated the utility for observing lower polygenic burden (PB) in large-effect variant (LEV) carriers than non-carriers, among cases (column S). We also estimated the number of LEV carriers per 1000 cases for different PB percentiles (columns T-AM).
