# Supplementary material for: Contextualizing genetic risk score for disease screening and rare variant discovery
Source: Nat Commun. 2021 Jul 20;12:4418. doi: 10.1038/s41467-021-24387-z (PMC8292385; doi:10.1038/s41467-021-24387-z)
Supplement: Supplementary file 6 — Reporting Summary [file 41467_2021_24387_MOESM6_ESM.pdf]

nature research

Corresponding author(s): Eric R. Gamazon

Last updated by author(s): Jun 5, 2021

Reporting Summary

Nature Research wishes to improve the reproducibility of the work that we publish. This form provides structure for consistency and transparency in reporting. For further information on Nature Research policies, see our Editorial Policies and the Editorial Policy Checklist.

Statistics

For all statistical analyses, confirm that the following items are present in the figure legend, table legend, main text, or Methods section.

n/a	Confirmed	
☐	☒	The exact sample size (*n*) for each experimental group/condition, given as a discrete number and unit of measurement
☐	☒	A statement on whether measurements were taken from distinct samples or whether the same sample was measured repeatedly
☐	☒	The statistical test(s) used AND whether they are one- or two-sided<br>*Only common tests should be described solely by name; describe more complex techniques in the Methods section.*
☐	☒	A description of all covariates tested
☐	☒	A description of any assumptions or corrections, such as tests of normality and adjustment for multiple comparisons
☐	☒	A full description of the statistical parameters including central tendency (e.g. means) or other basic estimates (e.g. regression coefficient) AND variation (e.g. standard deviation) or associated estimates of uncertainty (e.g. confidence intervals)
☐	☒	For null hypothesis testing, the test statistic (e.g. *F*, *t*, *r*) with confidence intervals, effect sizes, degrees of freedom and *P* value noted<br>*Give P values as exact values whenever suitable.*
☒	☐	For Bayesian analysis, information on the choice of priors and Markov chain Monte Carlo settings
☒	☐	For hierarchical and complex designs, identification of the appropriate level for tests and full reporting of outcomes
☐	☒	Estimates of effect sizes (e.g. Cohen's *d*, Pearson's *r*), indicating how they were calculated

Our web collection on statistics for biologists contains articles on many of the points above.

Software and code

Policy information about availability of computer code

Data collection	We have clarified the data sources in the "Data availability" section. The URLs and the DOI for accessing the datasets have been provided.
Data analysis	The scripts for the analyses are available on GitHub through the URL [https://github.com/gamazonlab/Polygenic_Background_Rare_Variant_Axis] included in the "Code availability". We also provided a permanent DOI for the source code (release version 1) and empirical results on Zenodo at http://doi.org/10.5281/zenodo.4767933. The publicly available software, PB-LEV-SCAN, is available on GitHub through the URL included in the "Code availability".

For manuscripts utilizing custom algorithms or software that are central to the research but not yet described in published literature, software must be made available to editors and reviewers. We strongly encourage code deposition in a community repository (e.g. GitHub). See the Nature Research guidelines for submitting code & software for further information.

Data

Policy information about availability of data

All manuscripts must include a data availability statement. This statement should provide the following information, where applicable:

- Accession codes, unique identifiers, or web links for publicly available datasets
- A list of figures that have associated raw data
- A description of any restrictions on data availability

The availability of data has been clarified in the "Data availability" section. The summary statistics on the UK Biobank are publicly available. The download link and the technical details can be found at http://www.nealelab.is/uk-biobank and http://www.nealelab.is/blog/2017/9/14/heritability-501-ldsr-based-h2-in-ukbb-for-the-technically-minded. The trait-associated variants from exome sequencing come from recent published studies[https://doi.org/10.1101/2020.12.13.422582][https://doi.org/10.1038/s41436-020-01007-7]. The summary statistics involving the MAF and LD-score data, which are used in the simulations, can be retrieved

from https://github.com/gamazonlab/Polygenic_Background_Rare_Variant_Axis (also available now with a permanent DOI on Zenodo at http://doi.org/10.5281/zenodo.4767933). All requests for raw BioVU data (for example, genotype) are reviewed by Vanderbilt University Medical Center to determine whether the request is subject to any intellectual property or confidentiality obligations. For example, patient-related data not included in the paper may be subject to patient confidentiality. Any such data and materials that can be shared will be released via a material transfer agreement. The simulation studies were performed using the software we created for this project (https://github.com/gamazonlab/Polygenic_Background_Rare_Variant_Axis). These simulations were informed by linkage disequilibrium patterns and allele frequency information from empirical data (BioVU) and the simulation parameters reflected empirical parameters from disease phenotype data (UK Biobank). Information on TS and OCD is available from the published GWAS of TS and OCD. GWAS summary statistics and data access for TS and OCD can be obtained from the Psychiatric Genomics Consortium website: https://www.med.unc.edu/pgc/pgc-workgroups/ocd-tourette-syndrome/. We analyzed the previously published WTCCC T1D dataset. WTCCC data access for T1D cases and controls is described at https://www.wtccc.org.uk/info/access_to_data_samples.html.

Field-specific reporting

Please select the one below that is the best fit for your research. If you are not sure, read the appropriate sections before making your selection.

☒ Life sciences ☐ Behavioural & social sciences ☐ Ecological, evolutionary & environmental sciences

For a reference copy of the document with all sections, see nature.com/documents/nr-reporting-summary-flat.pdf

Life sciences study design

All studies must disclose on these points even when the disclosure is negative.

Sample size	The simulations were performed based on the 23,294 European ancestry subjects. This sample is much larger than the sample size in the 1000 Genome Project, which is typically used to calculate these SNP-level statistics. To test the PB-LEV correlation, available empirical GWAS datasets (516, 919, and 1858 cases of TS, OCD, and T1D, respectively; the sample sizes for the UKB can be found the Supplementary Data 2) were used in this study.
Data exclusions	Samples with low genotyping or imputation quality were excluded. For this study, we included European ancestry subjects to avoid population stratification.
Replication	We applied our framework to 36 traits from the UKB. For the traits with available LEV data, the observed values in the UK Biobank were highly consistent with the predicted values from the framework, confirming the framework.
Randomization	This is not relevant to our study since it is not a randomized controlled trial.
Blinding	This is not relevant to our study since it is not a randomized controlled trial.

Reporting for specific materials, systems and methods

We require information from authors about some types of materials, experimental systems and methods used in many studies. Here, indicate whether each material, system or method listed is relevant to your study. If you are not sure if a list item applies to your research, read the appropriate section before selecting a response.

Materials & experimental systems

n/a	Involved in the study	
☒	☐	Antibodies
☒	☐	Eukaryotic cell lines
☒	☐	Palaeontology and archaeology
☒	☐	Animals and other organisms
☒	☐	Human research participants
☒	☐	Clinical data
☒	☐	Dual use research of concern

Methods

n/a	Involved in the study	
☒	☐	ChIP-seq
☒	☐	Flow cytometry
☒	☐	MRI-based neuroimaging
